# Thermostable Heptaplex PCR Assay for the Detection of Six Respiratory Bacterial Pathogens

**DOI:** 10.3390/diagnostics11050753

**Published:** 2021-04-22

**Authors:** Nik Mohd Noor Nik Zuraina, Mohammed Dauda Goni, Khazani Nur Amalina, Habsah Hasan, Suharni Mohamad, Siti Suraiya

**Affiliations:** 1 Department of Medical Microbiology and Parasitology, Universiti Sains Malaysia, Kota Bharu 16150, Kelantan, Malaysia; nikzurainanmn@gmail.com (N.M.N.N.Z.); namalinakhazani@gmail.com (K.N.A.); drhabsah@usm.my (H.H.); 2 Faculty of Veterinary Medicine, Universiti Malaysia Kelantan, Kota Bharu 16100, Kelantan, Malaysia; dauda.g@umk.edu.my; 3 School of Dental Sciences, Universiti Sains Malaysia, Kota Bharu 16150, Kelantan, Malaysia; suharni@usm.my

**Keywords:** *Klebsiella pneumoniae*, *Staphylococcus aureus*, *Streptococcus pneumoniae*, *Pseudomonas aeruginosa*, *Mycobacterium tuberculosis*, *Haemophilus influenzae*

## Abstract

A thermostabilized, multiplex polymerase chain reaction (mPCR) assay was developed in this study for the detection of six respiratory bacterial pathogens. Specific primers were designed for an internal amplification control (IAC) and six target sequences from *Klebsiella pneumoniae*, *Staphylococcus aureus*, *Streptococcus pneumoniae*, *Pseudomonas aeruginosa*, *Mycobacterium tuberculosis*, and *Haemophilus influenzae*. The resultant seven-band positive amplification control (PAC) of this heptaplex PCR assay corresponded to 105 base pairs (bp) of IAC, 202 bp of *K. pneumoniae*, 293 bp of *S. aureus*, 349 bp of *S. pneumoniae*, 444 bp of *P. aeruginosa*, 505 bp of *M. tuberculosis*, and 582 bp of *H. influenzae*. Results found that 6% (*w*/*v*) of the stabilizer was optimum to preserve the functional conformation of *Taq* DNA polymerase enzyme. This assay was stable at ambient temperature for at least 6 months. The sensitivity and specificity of this assay were both 100% when testing on the intended target organisms (*n* = 119) and non-intended species (*n* = 57). The mPCR assay developed in this study enabled accurate, rapid, and simple detection of six respiratory bacteria.

## 1. Introduction

The rules of thumb for treating any infectious diseases include early yet accurate diagnosis and appropriate antibiotic treatment [1,2]. Failure to meet these criteria could lead to serious health issues and increase the risk of morbidity and mortality [2]. Annually, respiratory tract infections (RTIs) have been constantly listed in the top 10 global causes of death by the World Health Organization (WHO). On top of communicable diseases in global, lower RTIs were the leading cause of death in 2019, followed by human immunodeficiency virus infection and acquired immune deficiency syndrome (HIV/AIDS), tuberculosis, and diarrheal diseases [3]. Out of 450 million people suffering from pneumonia annually, 4.2 million deaths of lower respiratory infections occur worldwide among all age groups with 1.8 million deaths are children between ages 1 to 59 months [4]. 

One of the strategies as described elsewhere to reduce the high mortality rate of RTIs is to improve the laboratory tools and techniques for detecting the pathogens. Common bacterial pathogens associated with RTIs include *Haemophilus influenzae*, *Klebsiella pneumoniae*, *Mycobacterium tuberculosis*, *Streptococcus pneumoniae*, *Pseudomonas aeruginosa*, and *Staphylococcus aureus* [5,6]. The current bacterial culture and biochemical tests as the gold-standard methods are relatively insensitive, time-consuming, and expensive [7]. Recently, various technologies and platforms have been introduced and applied in the development of molecular diagnostic tests for RTIs. These include liquid-chip-based assay for viral pathogens [8], qualitative and quantitative PCR assays for individual detection of *S. pneumoniae*, *H. influenzae*, *K. pneumoniae*, *S. aureus*, *P. aeruginosa*, and *M. tuberculosis* [9,10,11,12,13], agarose gel and capillary electrophoresis-based qualitative mPCR for unculturable viral and atypical bacterial pathogens [14,15], bead-based suspension array targeting 20 pathogens (viruses, atypical bacteria, *Acinetobacter baumannii*, *K. pneumoniae*, *S. aureus*, *S. pneumoniae*, *P. aeruginosa*, and *Stenotrophomonas maltophilia*) [16], microfluidic-based singleplex, real-time PCR using a TaqMan Array Card that can detect 32 pathogens (24 viruses, eight bacteria and two fungi) [17], and a paper/polymer hybrid microfluidic platform using loop-mediated isothermal amplification in the point-of care biochip for *B. pertussis* [18]. 

However, although some of the available diagnostic tests have a wide range of pathogen coverage and have been approved by the FDA (Food and Drug Administration), there are still several limitations, for instance, relatively longer hands-on time, costly, or laborious. For example, the TaqMan Array Card allows for a maximum of eight samples per run and requires expensive equipment. The paper/polymer hybrid microfluidic platforms are still new as diagnostic platforms and require further evaluations on clinical specimens for accuracy validation. Additionally, most of these available tests are meant for targeting respiratory viruses and atypical bacteria. 

Therefore, more reliable assays that are convenient, cost-effective, rapid, and effective are still needed. The present study describes a simplified, ready-to-use, dry-based mPCR assay targeting for *H. influenzae*, *K. pneumoniae*, *M. tuberculosis*, *S. pneumoniae*, *P. aeruginosa*, and *S. aureus,* using a panel of specific genes of the target bacteria. These genes include *K. pneumoniae*’s phosphohydrolase (designated as *php* for this study), *S. aureus*’s factor essential for methicillin (*fem*A), *S. pneumoniae*’s pneumolysin (*ply*), *P. aeruginosa*’s outer membrane lipoprotein (*opr*L), *M. tuberculosis*’s heat-shock protein (*hsp*65), and *H. influenzae*’s outer membrane protein (*omp*6). This thermostabilized, single-tube mPCR would be beneficial for the management of respiratory infections, especially under mass gatherings and other challenging conditions.

## 2. Materials and Methods

### 2.1. Bacterial Strains and Clinical Isolates

Reference bacterial strains as the source for developing the PAC were derived from the American Type Culture Collection (ATCC): *K. pneumoniae* ATCC BAA-1706, *S. aureus* ATCC 25923, *S. pneumoniae* ATCC 49619, *P. aeruginosa* ATCC 27853, *M. tuberculosis* H37Rv, and *H. influenzae* ATCC 49247. Other standard reference strains used for the sensitivity and specificity evaluation in this study includes *K. pneumoniae* ATCC BAA-1705, *S. aureus* ATCC 33591, *S. pneumoniae* ATCC 51916, *S. pneumoniae* ATCC 700673, *P. aeruginosa* ATCC 9027, *Mycobacterium bovis* ATCC 35720, *H. influenzae* ATCC 49766, *Acinetobacter baumannii* ATCC 19606, *Aeromonas hydrophila* ATCC 7966T, *Bacillus cereus ATCC 14579*, *Bacillus subtilis ATCC 6633*, *Enterobacter aerogenes* *ATCC 13048*, *Enterobacter cloacae* ATCC 13047, *Escherichia coli* ATCC 25922, *E. coli* O157 non-toxigenic NCTC 12900, *Listeria monocytogenes* ATCC 7644, *Neisseria meningitidis* ATCC 13090, *Neisseria gonorrhoeae* ATCC 43069, *Proteus mirabilis* ATCC 29245, *Staphylococcus epidermidis* ATCC 12228, *Streptococcus viridians* ATCC 36395, *Streptococcus pyogenes* ATCC 19615, *Streptococcus mutans* ATCC 35668, and *Streptococcus sanguinis* ATCC 10556. A total of 129 clinical isolates were acquired from the Department of Medical Microbiology and Parasitology, Universiti Sains Malaysia (USM), Malaysia. 

### 2.2. Primers

Seven pairs of primers were designed for the target bacteria and IAC using the National Center for Biotechnology Information (NCBI) primer Basic Local Alignment Search Tool (BLAST). The expected PCR amplicons were distinct from one another by approximately 50 to 100 bp. The primers were synthesized by Integrated DNA Technologies, Singapore. Each of the primers was initially subjected to in silico analyses for checking the specificity and the presence of secondary structure, which were done through the NCBI nucleotide BLAST program (https://blast.ncbi.nlm.nih.gov/Blast.cgi; accessed on: 8 June 2017) and through the Oligo Analyzer online tool (https://sg.idtdna.com/calc/analyzer; accessed on: 21 September 2017), respectively. All the primers were also tested individually using monoplex PCR over the same target species and other bacterial isolates. The constituted primers were diluted to 20 µM and stored at −20 °C. The primer sequences are shown in Table 1.

### 2.3. Preparation of PAC and IAC Templates

The seven-band PAC was constructed to ensure that the primers and other PCR components were working well during PCR amplification. In addition to PAC, an IAC, which was designed from the phosphoglucosamine mutase (*glmM*) gene of *H. pylori*, was incorporated to rule out PCR inhibition. Bacterial DNA for the construction of PAC and IAC was extracted from ATCC strains using a commercialized DNA extraction kit by following the manufacturer’s guidelines (Qiagen GmbH, Hilden, Germany). The gene fragments of *glm*M, *K. pneumoniae*’s *php*, *S. aureus*’s *fem*A, *S. pneumoniae*’s *ply*, *P. aeruginosa*’s *opr*L, *M. tuberculosis*’s *hsp*65, and *H. influenzae*’s *omp*6 were cloned individually into a pCR2.1-TOPO^®^ plasmid according to the manufacturer’s guidelines (Invitrogen, Beverly, MA, USA). Each cloned plasmid was verified by PCR and DNA sequencing and was diluted to 1 ng/µL. The cloned plasmids (excluding IAC template) were mixed together as the PAC template. The final concentration of each plasmid was 10 pg/µL in a total volume of 20 µL PCR reaction.

### 2.4. Preparation of DNA Templates from Clinical Isolates

DNA from the clinical isolates was prepared using the boiling method. Briefly, a loopful of colony from each clinical isolate was suspended into 100 µL of autoclaved distilled water and boiled for 10 min. The intracellular component containing nucleic acid was separated from cell-wall debris by high-speed centrifugation at 10,000× *g* for 5 min. The supernatant was used as DNA template for this heptaplex PCR evaluation.

### 2.5. Development of Heptaplex PCR Assay

A standard monoplex PCR was performed in a total volume of 20 µL containing 1 × PCR buffer (Bioline, Boston, MA, USA), 2.5 mM magnesium chloride (MgCl_2_) (Bioline GmbH, London, UK), 0.2 mM deoxynucleotides (dNTPs) (Bioline GmbH, London, UK), 1 µM of each sense and antisense primer, and 0.75 units of *Taq* DNA polymerase enzyme (Bioline GmbH, London, UK). The cycling conditions used in this study consisted of initial denaturation at 95 °C (5 min), 30 cycles of denaturation at 95 °C (30 s), annealing at 60 °C (30 s), elongation at 72 °C (30 s), and a final elongation at 72 °C (10 min). On the basis of the conditions used in monoplex PCR, the components for heptaplex PCR were optimized individually to ensure that all the target DNA could be amplified simultaneously. Initially, different concentrations of primer mixture ranging from 0.2 to 0.8 µM were prepared and tested on the PAC template. Following the primer optimization, dNTPs were tested between 0.1 to 0.3 mM. Different concentrations of MgCl_2_ were also tested, where the final concentrations ranged between 1.5 mM and 3.5 mM. Subsequently, *Taq* DNA polymerase enzyme was optimized in a range of 0.5 to 1.5 units. PCR products were separated by electrophoresis at 90 volts for 60 min on 1.5% agarose gel and stained with FloroSafe DNA stain (1^ST^ BASE, Singapore Science Park II, Singapore). 

### 2.6. Thermostabilization and Stability Evaluation of the Heptaplex PCR Assay

Thermostabilization of this heptaplex PCR assay was done through a total dehydration of the PCR components. The sugar surfactant enzyme stabilizer, trehalose anhydrous, was used to preserve the functional conformation of the *Taq* polymerase enzyme during the dehydration process. The concentration of trehalose was tested at 2%, 4%, 6%, 8%, and 10% (*w*/*v*). After deglycerolizing the *Taq* DNA polymerase enzyme using the spin-column method, the PCR mixture then underwent a freeze-drying process using a vacuum centrifuge lyophilizer [8,9]. The performance of thermostabilized heptaplex PCR assay was evaluated at different temperatures of 4 °C, 25 °C, 37 °C, and 45 °C for 60 days.

### 2.7. Analytical Sensitivity and Specificity Evaluation of the Heptaplex PCR Assay

The single-tube PCR assay was initially evaluated on the reference strains from both intended (*n* = 13) and non-intended (*n* = 15) target bacteria. The sensitivity of this PCR was also evaluated using a total of 98 intended target clinical isolates: *K. pneumoniae* (*n* = 17)*, S. aureus* (*n* = 17), *S. pneumoniae* (*n* = 17), *P. aeruginosa* (*n* = 17)*, M. tuberculosis* (*n* = 16), and *H. influenzae* (*n* = 17). Additionally, a total of 31 unknown clinical isolates were used for further accuracy performance in a randomized, blind test. The clinical isolates were obtained from various sources of clinical samples including blood, sputum, body fluids, and swabs. All the clinical isolates were identified using the gold standard bacterial culture methods and VITEK 2 System (BioMérieux, Craponne, France) in a routine diagnostic laboratory. To summarize, a total of 157 bacteria, comprising both reference strains (*n* = 28) and clinical isolates (*n* = 129), were used. Two microliters of DNA prepared from each isolate was used as DNA template in the evaluation of the heptaplex PCR assay.

## 3. Results

### 3.1. Development of the Heptaplex PCR Assay

**Figure 1:**
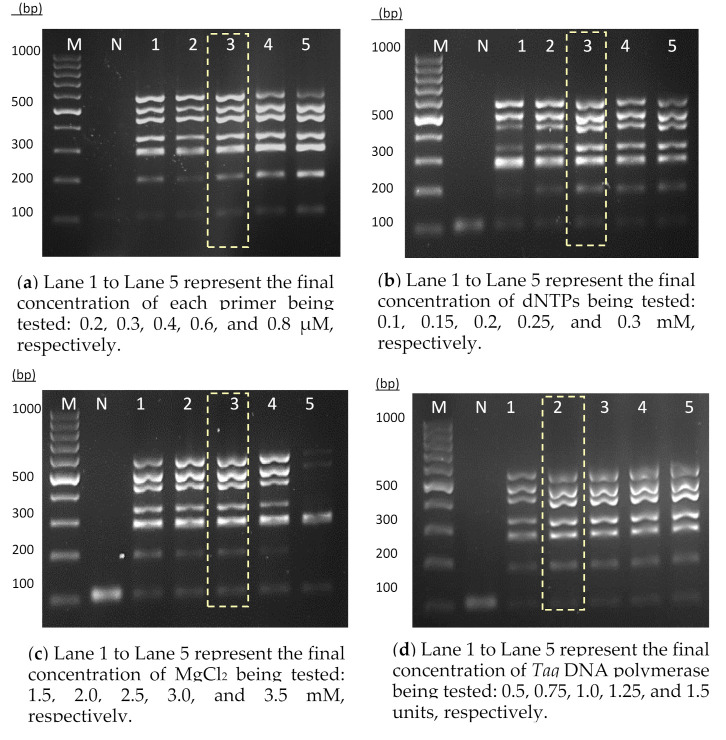
Optimization of the PCR components for the heptaplex PCR assay: (**a**) primers, (**b**) dNTPs, (**c**) MgCl_2_, and (**d**) *Taq* DNA polymerase enzyme. Lane M: 100 bp DNA ladder (Fermentas, Provincetown, MA, USA); Lane N: IAC; Lanes 1–5 are the corresponding amplicons of targeted genes under different conditions. The highlighted lanes represent the optimal concentrations of primers, dNTPs, MgCl_2_, and *Ta* DNA polymerase used for this assay.

A thermostabilized single-tube heptaplex PCR assay incorporated with IAC was developed for the detection of six respiratory bacterial pathogens. The primers designed for this heptaplex PCR showed a high degree of specificity through in silico analysis. Each primer sequence was analyzed over 40 billion sequences available in the nucleotide collection of the NCBI database. There was no cross-reactivity observed between all the intended target panels nor across other organisms including RTI-related pathogens and human. Additionally, the primer sequences were noted to have an acceptable value of delta G (around −3 kcal/mol). A larger negative value of delta G (>−9 kcal/mol) would lead to the formation of primer secondary structures. The melting temperatures (below 10 °C) of possible hairpin structures were also far lower than the reaction annealing temperature (60 °C). One primer pair was used to amplify one specific target DNA. During the optimization stage, different concentrations of primers, dNTPs, MgCl_2_, and *Taq* DNA polymerase were tested. On the basis of the results (Figure 1), the final concentrations of 0.4 µM of each primer (Figure 1a), 0.2 mM dNTPs (Figure 1b), 2.5 mM MgCl_2_ (Figure 1c), and 0.75 units of *Taq* DNA polymerase enzyme (Figure 1d) were selected as the optimum concentration to amplify the corresponding target DNA uniformly. 

### 3.2. Thermostabilization and Stability Evaluation of the Heptaplex PCR Assay

**Figure 2:**
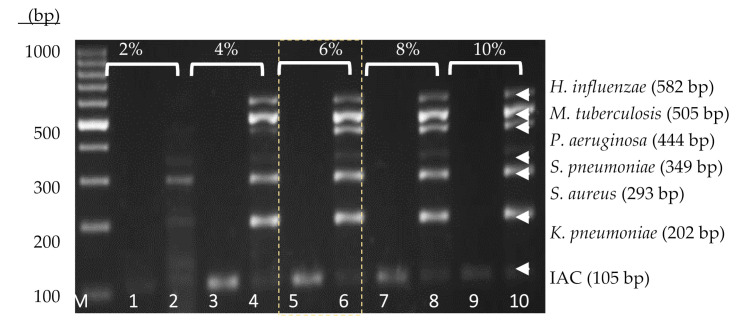
Agarose gel electrophoresis image for the optimization of trehalose enzyme stabilizer (2%, 4%, 6%, 8%, and 10%) along with the 100 bp DNA ladder. Lane 1 to Lane 10: the respective IACs and PACs at different concentrations of trehalose, ranging from 2% to 10%. The use of 6% trehalose was selected for this heptaplex PCR assay.

A thermostabilized PCR reagent is achieved through total dehydration of the PCR components in the presence of enzyme stabilizer. In this study, the concentration of trehalose anhydrous was optimized, and 6% (*w*/*v*) (Figure 2) was selected for further use in the development of the thermostabilized heptaplex PCR assay. 

**Figure 3:**
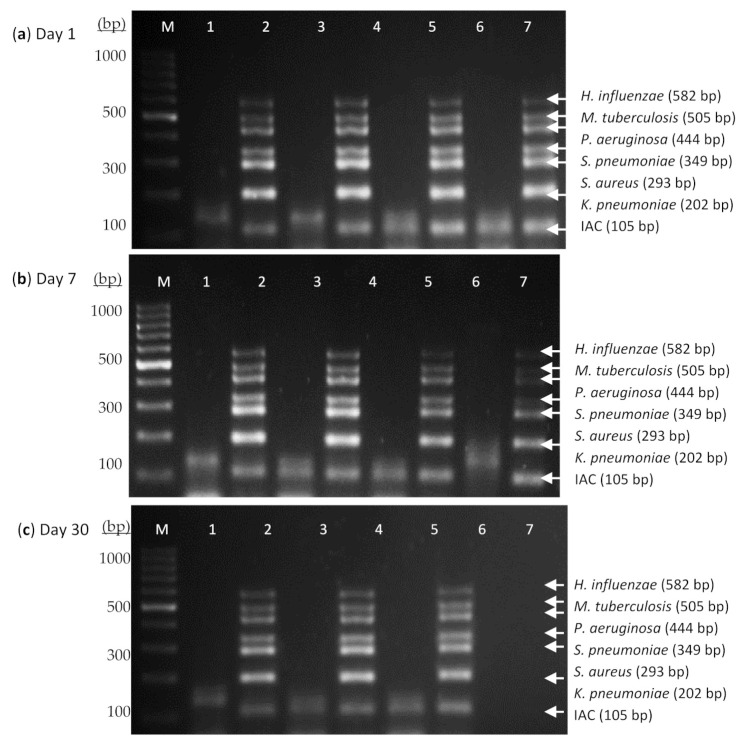

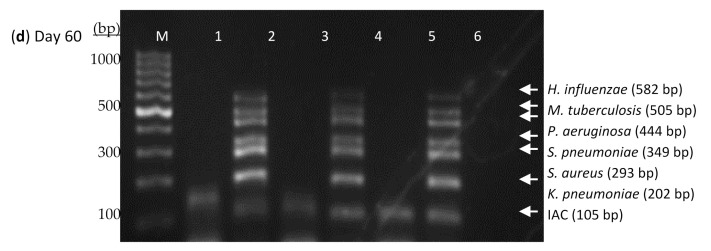
Stability evaluation of thermostabilized heptaplex PCR assay in the presence of 6% trehalose at 4 °C, 25 °C, 37 °C, and 45 °C on (**a**) Day 1, (**b**) Day 7, (**c**) Day 30, and (**d**) Day 60. Lane M: 100 bp DNA ladder; Lane 1: IAC at 4 °C; Lane 2: PAC at 4 °C; Lane 3: IAC at 25°C; Lane 4: PAC at 25 °C; Lane 5: IAC at 37 °C; Lane 6: PAC at 37 °C; Lane 7: IAC at 45 °C; Lane 8: PAC at 45 °C. The dry-based PCR reagent was found to be stable at 4 °C, 25 °C, and 37 °C for at least 60 days as tested in this study.

Shelf-life is the period for which the reagent functions appropriately. The stability of this assay was evaluated at different temperatures for a total duration of 60 days. Results showed that the thermostabilized PCR was stable at 4 °C, 25 °C, and 37 °C when tested at different day intervals (Figure 3). 

Using 37 °C as an elevated temperature and 60 days total length of time, the shelf-life of this PCR assay was calculated using the Q_10_ formula according to Clark (1991) [10]. As referenced in Clark (1991), the acceleration factor of 10 °C rise (Q_10_) is 1.8.

The acceleration factor (AF) of this shelf-life test was calculated as follows:

AF = Q_10_^[0.1 × (T_e_ − T_a_)],

where AF is the acceleration factor, Q_10_ = 1.8, T_e_ is the elevated temperature (37 °C), and T_a_ is the ambient temperature (25 °C). Thus, AF = 1.8^[0.1 × (37 − 25) = 2.025. The acceleration factor of this shelf life test was 2.025.

The accelerated age was calculated as follows:

AG = *t*_e_ × AF,

where AG is the accelerated age and *t*_e_ is the length of time at elevated temperature (60 days). Thus, AG = 60 × 2.025 = 121 days. The accelerated age was calculated as 121 days.

The estimated shelf life was calculated as follows:

Estimated shelf life = AG + *t*_e_,

where AG is the accelerated age (121 days), and *t*_e_ is the length of time at elevated temperature (60 days). Thus, estimated shelf life = 121 + 60 = 181 days. The estimated shelf life for this assay was calculated as 181 days, which is equal to 6 months.

### 3.3. Sensitivity and Specificity Evaluation of the Heptaplex PCR on the Reference Strains and Clinical Isolates

**Figure 4:**
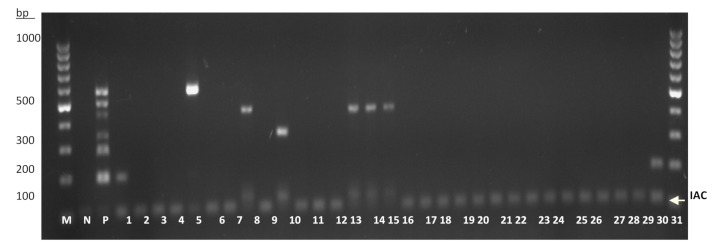
A randomized, blind test for the accuracy performance of the single-tube heptaplex PCR on clinical isolates (*n* = 31). The presence of amplicon bands on the agarose gel corresponds to the respective target organisms, whereby the accuracy performance of this assay was compared to the blinded test. Lane M: 100 bp DNA ladder, Lane N: IAC, Lane P: PAC, Lane 1: *K. pneumoniae*, Lane 2: *Acinetobacter* species, Lane 3: *Achromobacter xylosoxidans*, Lane 4: *Moraxella catarrhalis*, Lane 5: *H. influenzae*, Lane 6: *E. coli*, Lane 7: *Citrobacter freundii*, Lane 8: *P. aeruginosa*, Lane 9: *Streptococcus* group A, Lane 10: *S. pneumoniae*, Lane 11: *E. coli*, Lane 12: *E. coli*, Lane 13: *Streptococcus* group B, Lane 14: *P. aeruginosa*, Lane 15: *P. aeruginosa*, Lane 16: *P. aeruginosa*, Lane 17: *Streptococcus* group B, Lane 18: *Enterobacter* species, Lane 19: *A. xylosoxidans*, Lane 20: *Streptococcus* group A, Lane 21: *Acinetobacter baumannii*, Lane 22: *Streptococcus* group G, Lane 23: *Streptococcus* group C, Lane 24: *Streptococcus* group B, Lane 25: *E. coli*, Lane 26: *Streptococcus viridans*, Lane 27: *Serratia marcescens*, Lane 28: *Roseomonas gilardii*, Lane 29: *Streptococcus* group A, Lane 30: *Acinetobacter* species, and Lane 31: *K. pneumoniae*.

The single-tube heptaplex PCR assay was further evaluated for its sensitivity and specificity on reference bacterial strains and clinical isolates. Sensitivity can be defined as the proportion of positive specimens that tested positive, while specificity conversely means the proportion of negative specimens that tested negative [11]. In addition, the test accuracy refers to the proportion of all specimens that were correctly classified among all subjects [11]. For the initial test accuracy using reference bacterial strains, all the intended targets (*n* = 13) were successfully amplified (Figure S1a, Supplementary Materials) while no amplicon was observed among the non-intended target strains (*n* = 34) (Figure S1b,c, Supplementary Materials). Therefore, the results indicated that the preliminary sensitivity and specificity for this assay were both 100%. In the subsequent sensitivity test, the heptaplex PCR successfully detected all 98 samples of intended bacterial isolates (Figure S2a–f, Supplementary Materials). In addition, this assay successfully detected the intended target organisms (*n* = 8) in addition to the non-intended organisms (*n* = 23), as shown by the presence or absence of amplicons in a randomized, blind test (Figure 4), in which the specific organisms used for this blind test were initially unknown. Overall, the results showed that this heptaplex PCR was 100% sensitive (95% confidence interval: 96.95% to 100%) over the intended isolates (*n* = 119) and 100% specific (95% confidence interval: 93.73% to 100%) over the non-intended isolates (*n* = 57) (Table 2).

## 4. Discussion

This study describes the development of a thermostabilized single-tube heptaplex PCR targeting six bacterial pathogens: *K. pneumoniae*, *S. aureus*, *S. pneumoniae*, *P. aeruginosa*, *M. tuberculosis*, and *H. influenzae*. These bacteria are important as the etiological agents of RTIs. *H. influenzae* and *S. pneumoniae* have been the two predominant bacteria responsible for community-acquired pneumonia (CAP). Meanwhile, *K. pneumoniae*, *S. aureus*, *P. aeruginosa*, and *M. tuberculosis* are known to be associated with severe RTIs, especially when the causal strains are antibiotic-resistant. For instance, it has been reported that the mortality rate for CAP due to *S. aureus* (9.1% to 13.3%) has been found higher than that of the pneumococcal (4.4%) or other etiologic bacteria (2.0%) in the USA [12].

The developed mPCR assay of this study consisted of seven pairs of primers, *Taq* DNA polymerase, enzyme stabilizer, and other PCR components in its dry-based formulation. Under a series of optimization steps, seven target amplicons ranging from 105 bp to 582 bp were developed for the PAC. All the primers were designed to have uniform GC content and melting temperature. These conditions enabled amplification of all the respective bacterial DNA at the same annealing temperature. In addition to a PAC, an IAC was incorporated in this assay and was constructed from the *glm*M gene of *H. pylori*, a non-respiratory bacterial pathogen. This gene is highly recommended for molecular diagnosis of *H. pylori* infection due to its specificity [13]. Therefore, it was applied in the present work for the development of a specific IAC that has no cross-reactivity with other intended and non-intended targets. Incorporation of an IAC in this mPCR assay could validate a successful PCR amplification. The absence of this IAC band would indicate the presence of PCR inhibitors that can lead to false-negative results [14]. 

It is well noted that setting up monoplex PCR reactions for detecting different organisms at one time is tedious. Using this heptaplex PCR assay, detection of the panel bacterial pathogens in clinical diagnoses would be easier and faster by simply rehydrating the dry-based reagent and adding DNA sample. As a preoptimized master mix PCR kit, this heptaplex PCR assay could eliminate the requirement of tedious optimization and calculation, as well as minimize the pipetting steps, much like in conventional PCR. Hence, it may shorten the duration of PCR setup and reduce contamination. The results can be obtained in approximately 4 h, starting from the DNA sample preparation to the final gel interpretation. The heptaplex PCR assay developed in this study revealed its high accuracy when tested on a number of bacterial isolates. It, hence, enables sensitive detection of *K. pneumoniae*, *S. aureus*, *S. pneumoniae*, *P. aeruginosa*, *M. tuberculosis*, and *H. influenzae* in clinical samples. The single-tube assay permitted amplification of the IAC and at least one targeted respiratory bacterium. Likewise, the mPCR assay can detect multiple bacteria simultaneously when more than one target bacterium is present in the sample. 

Its dry-based formula allows this assay to be stored and run at ambient temperature without the need for cold-chain transportation and storage. The stability evaluation of this assay showed that the thermostabilized heptaplex PCR was stable in a wide range of temperature, such as 4 °C (cold), 25 °C (ambient), and 37 °C (warm). According to the previous procedure used for developing thermostabilized PCR assays [8,15], optimization was done to ensure that all the freeze-dried PCR components worked well for this present study. Using the elevated temperature and the total length of tested stability period, the minimum shelf-life of this assay was found to be 6 months at ambient temperature. According to the mathematical Q_10_ formula, a longer tested stability period would result in a longer shelf-life being obtained. A long shelf-life of diagnostic PCR at ambient temperature is desirable for minimizing cost, warehousing, and end-user storage of the product [16].

This study showed that this PCR-based assay is a promising tool for the clinical diagnoses of RTIs. However, despite its high accuracy, one should note that the heptaplex PCR in the current study was evaluated merely on the pure colonies. In previous studies, problems in accuracy performance were reported when the PCR assay was tested on clinical specimens [17,18,19]. The reasons include a false-positive reaction due to the presence of contaminating DNA [20]. Moreover, clinical specimens are known to contain inhibitory substances that could lead to false-negative results [18]. Competition of primers is another drawback of mPCR, especially when more than one etiological bacterium is present [21]. Although the primers used in this study were shown to have great performance through both in silico and laboratory-based analyses, further study is still required to validate the reproducibility of this assay on clinical respiratory specimens such as sputum, pharyngeal swabs, and tracheal aspirates.

## 5. Conclusions

In conclusion, the heptaplex PCR developed in this study enables the detection of multiple target bacteria in one single tube. This assay is applicable for the laboratory diagnosis of common bacteria associated with RTIs, especially for patients infected with more than one bacterial pathogen. The thermostabilized, single-tube mPCR assay developed in this study may have advantages such as its rapidity, simplicity, accuracy, and cold-chain free. These are among important criteria for a diagnostic application during mass gatherings, as well as in rural, famine, and disaster areas.

## Figures and Tables

**Table 1:** Characteristics of the primers used for the development of heptaplex PCR assay.

Primers	Sequence (5′ → 3′)	Target Amplicon	Size (bp)
IAC_F 1AC_R	F: AAC TTA TCC CCA ATC GCG CA R: GCC CTT TCT TCT CAA GCG GT	*H. pylori* (IAC)	105
1_F 2_R	F: ATT TCT CCG GCG TCA AGT GT R: CTC AAC ATC GTC GCA AAG GC	*K. pneumoniae*	202
3_F 4_R	F: CGC AAA CTG TTG GCC ACT AT R: CTC GCC ATC ATG ATT CAA GT	*S. aureus*	293
5_F 6_R	F: TTG ACC CAT CAG GGA GAA AG R: CTT GAT GCC ACT TAG CCA AC	*S. pneumoniae*	349
7_F 8_R	F: GAT GGA AAT GCT GAA ATT CG R: GGA CGC TCT TTA CCA TAG GA	*P. aeruginosa*	444
9_F 10_R	F: TGG AGG ATC CGT ACG AGA AG R: TTG ACA GTG GAC ACC TTG GA	*M. tuberculosis* complex	505
11_F 12_R	F: GCG AAA GTC CAA GCC TCT CT R: TCA CCG TAA GAT ACT GTG CCT	*H. influenzae*	582

F (forward/sense sequence), R (reverse/anti-sense sequence), IAC (internal amplification control), bp (base pair).

**Table 2:** Specificity evaluation of the single-tube heptaplex PCR assay using VITEK 2 identified clinical isolates (*n* = 129).

	Organism	Reference Strains, *n*	Clinical Isolates, *n*	Total Test, *n*	Positive Test, *n* (%)	Negative Test, *n* (%)
1.	*K. pneumoniae*	2	19	21	21 (100)	0 (0)
2.	*S. aureus*	2	16	18	18 (100)	0 (0)
3.	*S. pneumoniae*	3	17	20	20 (100)	0 (0)
4.	*P. aeruginosa*	2	21	23	23 (100)	0 (0)
5.	*M. tuberculosis*	2	16	18	18 (100)	0 (0)
6.	*H. influenzae*	2	17	19	19 (100)	0 (0)
7.	*A. hydrophila*	1	0	1	0 (0)	1 (100)
8.	*A. baumannii*	1	2	3	0 (0)	3 (100)
9.	*Acinetobacter* spp.	0	3	3	0 (0)	3 (100)
10.	*A. xylosoxidans*	0	2	2	0 (0)	2 (100)
11.	*B. cereus*	1	0	1	0 (0)	1 (100)
12	*B. subtilis*	1	0	1	0 (0)	1 (100)
13.	*B. pseudomallei*	0	1	1	0 (0)	1 (100)
14.	*C. freundii*	0	2	2	0 (0)	2 (100)
15.	*Enterobacter* species	0	2	2	0 (0)	2 (100)
16.	*E. aerogenes*	1	0	1	0 (0)	1 (100)
17.	*E. cloacae*	1	0	1	0 (0)	1 (100)
18.	*E. coli*	1	6	7	0 (0)	7 (100)
19.	*E. coli* O157	1	0	1	0 (0)	1 (100)
20.	*Klebsiella* spp.	0	1	1	0 (0)	1 (100)
21.	*L*. *monocytogenes*	1	0	1	0 (0)	1 (100)
22.	*M. catarrhalis*	0	2	2	0 (0)	2 (100)
23.	*N. gonorrhoeae*	1	0	1	0 (0)	1 (100)
24.	*N. meningitidis*	1	0	1	0 (0)	1 (100)
25.	*P. mirabilis*	1	0	1	0 (0)	1 (100)
26.	*R. gilardii*	0	1	1	0 (0)	1 (100)
27.	*S. epidermis*	1	0	1	0 (0)	1 (100)
28.	*S. marcescens*	0	1	1	0 (0)	1 (100)
29.	*S. mutans*	1	0	1	0 (0)	1 (100)
30.	*S. pyogenes*	1	0	1	0 (0)	1 (100)
31.	*S. sanguinis*	1	0	1	0 (0)	1 (100)
32.	*S. viridians*	1	2	3	0 (0)	3 (100)
33.	*Serratia marcescens*	0	1	1	0 (0)	1 (100)
34.	*Streptococcus* group A	0	5	5	0 (0)	5 (100)
35.	*Streptococcus* group B	0	4	4	0 (0)	4 (100)
36.	*Streptococcus* group C	0	2	2	0 (0)	2 (100)
37.	*Streptococcus* group G	0	2	2	0 (0)	2 (100)
	Evaluation of the developed multiplex PCR assay for RTIs:					
	Total test, *N*		:	175		
	Total positive		:	119/119	(100%)	
	Total negative		:	57/57	(100%)	
	Test accuracy		:	175/175	(100%)	

## Supplementary Materials

The following are available online at https://www.mdpi.com/article/10.3390/diagnostics11050753/s1, Figure S1. Sensitivity and specificity evaluation of heptaplex PCR assay on (a) intended targets (*n* = 13), (b) non-intended targets (*n* = 17) from the reference strains, and (c) non-intended targets from clinical isolates (*n* = 17); Figure S2. Sensitivity evaluation of the single-tube heptaplex PCR on clinical isolates (*n* = 98): (a) *K. pneumoniae*, (b) *S. aureus*, (c) *S. pneumoniae*, (d) *P. aeruginosa*, (e) *M. tuberculosis*, and (f) *H. influenzae*.
