# Supplementary material for: Thermostable Heptaplex PCR Assay for the Detection of Six Respiratory Bacterial Pathogens
Source: Diagnostics (Basel). 2021 Apr 22;11(5):753. doi: 10.3390/diagnostics11050753 (PMC8146555; doi:10.3390/diagnostics11050753)
Supplement: Supplementary file 1 [file diagnostics-11-00753-s001.zip › diagnostics-1173611-supplementary.pdf]

Supplementary Materials

Figure S1. Sensitivity and specificity evaluation of heptaplex PCR assay on (a) intended targets (*n* = 13); (b) non-intended targets (*n* = 17) from the reference strains; and (c) non-intended targets from clinical isolates (*n* = 17).

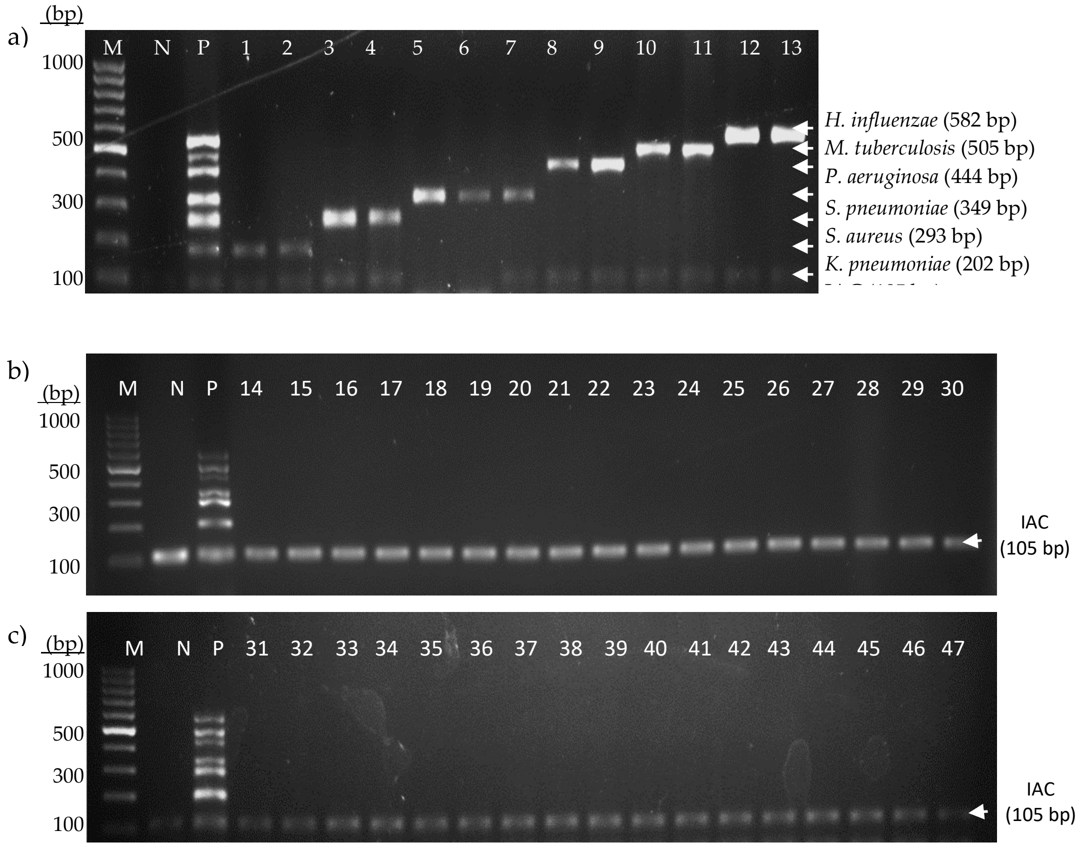

Lane M: 100 bp DNA ladder, Lane N: IAC, Lane P: PAC, Lane 1: *K. pneumoniae* ATCC 1706, Lane 2: *K. pneumoniae* ATCC 1705, Lane 3: *S. aureus* ATCC 25923, Lane 4: *S. aureus* ATCC 33591, Lane 5: *S. pneumoniae* ATCC 49619, Lane 6: *S. pneumoniae* ATCC 51916, Lane 7: *S. pneumoniae* ATCC 700673, Lane 8: *P. aeruginosa* ATCC 27853, Lane 9: *P. aeruginosa* ATCC 9027, Lane 10: *M. tuberculosis* H37Rv, Lane 11: *M. bovis* ATCC 35720, Lane 12: *H. influenzae* ATCC 49247, Lane 13: *H. influenzae* ATCC 49766, Lane 14: *A. hydrophila* ATCC 7966T, Lane 15: *Acinetobacter baumannii* ATCC 19606, Lane 16: *B. cereus* ATCC 14579, Lane 17: B. subtilis ATCC 6633, Lane 18: *E. aerogenes* ATCC 13048, Lane 19: *E. cloacae* ATCC 13047, Lane 20: *E. coli* ATCC 25922, Lane 21: *E. coli* O157 NCTC 12900, Lane 22: L. monocytogenes ATCC 7644, , Lane 23: *N. gonorrhoeae* ATCC 43069, Lane 24: *N. meningitidis* ATCC 13090, Lane 25: *P. mirabilis* ATCC 29245, Lane 26: *S. epidermidis* ATCC 12228, Lane 27: *S. mutans* ATCC 35668, Lane 28: *S. pyogenes* ATCC 19615, Lane 29: *S. sanguinis* ATCC 10556, Lane 30: *S. viridians* ATCC 36395, Lane 31: *Acinetobacter* spp., Lane 32: *A. xylosoxidans*, Lane 33: *A. baumannii*, Lane 34: *B. pseudomallei*, Lane 35: *C. freundii*, Lane 36: *Enterobacter* spp., Lane 37: *E. coli*, Lane 38: *E. coli*, Lane 39: *Klebsiella* spp., Lane 40: *M. catarrhalis*, Lane 41: *S. marcescens*, Lane 42: *Streptococcus* group A, Lane 43: *Streptococcus* group A, Lane 44: *Streptococcus* group B, Lane 45: *Streptococcus* group C, Lane 46: *Streptococcus* group G, and Lane 47: *S. viridans.*

Figure S2. Sensitivity evaluation of the single-tube heptaplex PCR on clinical isolates (*n* = 98): (a) *K. pneumoniae,* (b) *S. aureus,* (c) *S. pneumoniae,* (d) *P. aeruginosa,* (e) *M. tuberculosis* and (f) *H. influenzae*.

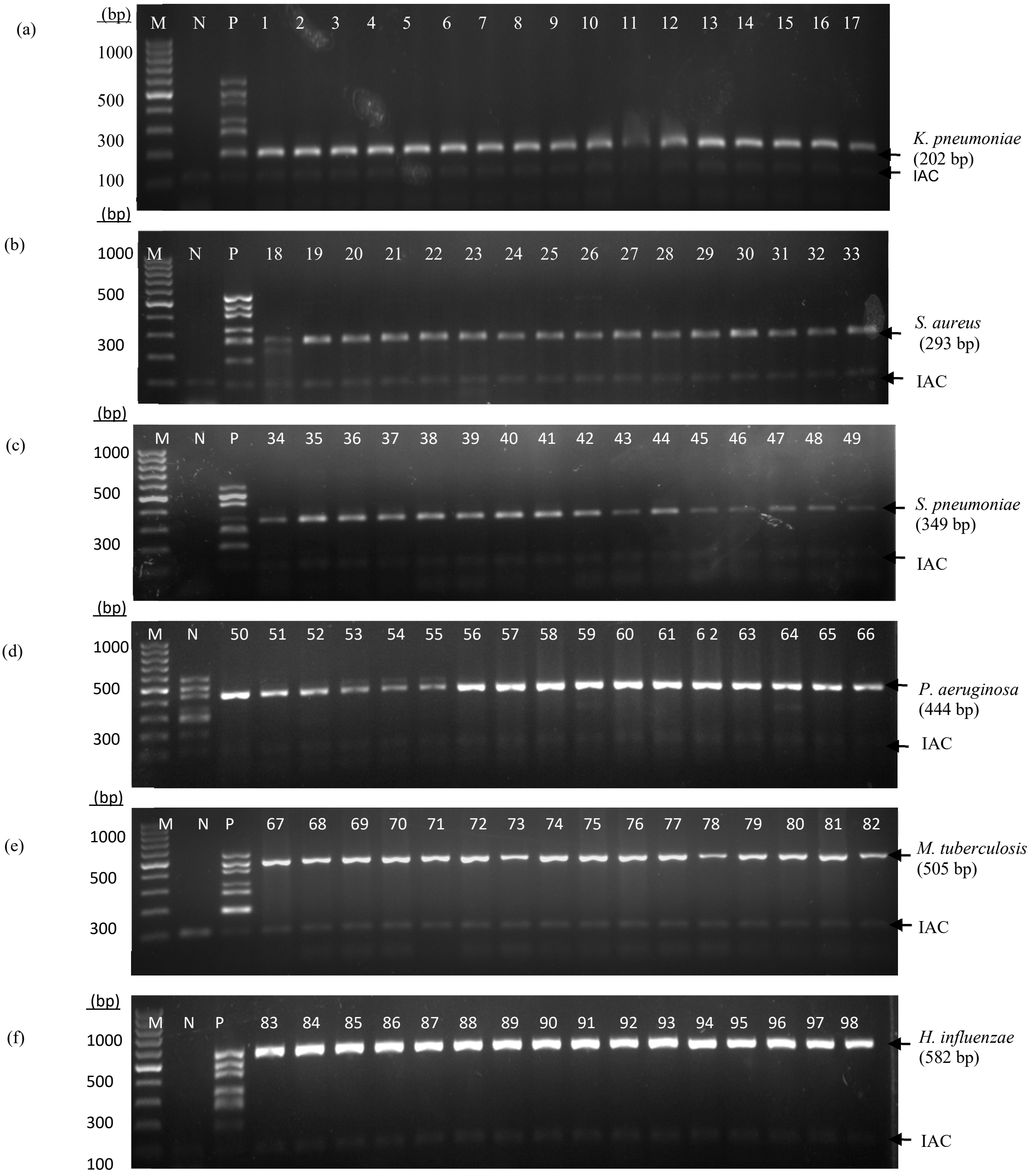

The developed assay had successfully detected all the intended target clinical isolates (n=98). Lane M: 100 bp DNA ladder, Lane N: IAC, Lane P: PAC, Lane 1 to Lane 17: amplicons of *K. pneumoniae* isolates, Lane 18 to Lane 33: amplicons of *S. aureus* isolates, Lane 34 to Lane 49: amplicons of *S. pneumoniae* isolates, Lane 50 to Lane 66: amplicons of *P. aeruginosa* isolates, Lane 67 to Lane 82: amplicons of *M. tuberculosis* isolates, Lane 83 to Lane 98: amplicons of *H. influenzae* isolates.
